# Novel Amphiphilic Block Copolymers for the Formation of Stimuli-Responsive Non-Lamellar Lipid Nanoparticles

**DOI:** 10.3390/molecules26123648

**Published:** 2021-06-15

**Authors:** Jiali Zhai, Bo Fan, San H. Thang, Calum J. Drummond

**Affiliations:** 1 School of Science, STEM College, RMIT University, Melbourne, VIC 3000, Australia; 2 School of Chemistry, Monash University, Clayton, VIC 3800, Australia; bo.fan@monash.edu (B.F.); san.thang@monash.edu (S.H.T.)

**Keywords:** monoolein, cubosome, RAFT, PDMAEMA, responsive nanoparticles, drug delivery, lyotropic liquid crystals, amphiphile block copolymer

## Abstract

Non-lamellar lyotropic liquid crystalline (LLC) lipid nanoparticles contain internal multidimensional nanostructures such as the inverse bicontinuous cubic and the inverse hexagonal mesophases, which can respond to external stimuli and have the potential of controlling drug release. To date, the internal LLC mesophase responsiveness of these lipid nanoparticles is largely achieved by adding ionizable small molecules to the parent lipid such as monoolein (MO), the mixture of which is then dispersed into nanoparticle suspensions by commercially available poly(ethylene oxide)–poly(propylene oxide) block copolymers. In this study, the Reversible Addition-Fragmentation chain Transfer (RAFT) technique was used to synthesize a series of novel amphiphilic block copolymers (ABCs) containing a hydrophilic poly(ethylene glycol) (PEG) block, a hydrophobic block and one or two responsive blocks, i.e., poly(4-(4,4,5,5-tetramethyl-1,3,2-dioxaborolan-2-yl)benzyl acrylate) (PTBA) and/or poly(2-(dimethylamino)ethyl methacrylate) (PDMAEMA). High throughput small angle X-ray scattering studies demonstrated that the synthesized ABCs could simultaneously stabilize a range of LLC MO nanoparticles (vesicles, cubosomes, hexosomes, inverse micelles) and provide internal particle nanostructure responsiveness to changes of hydrogen peroxide (H_2_O_2_) concentrations, pH and temperature. It was found that the novel functional ABCs can substitute for the commercial polymer stabilizer and the ionizable additive in the formation of next generation non-lamellar lipid nanoparticles. These novel formulations have the potential to control drug release in the tumor microenvironment with endogenous H_2_O_2_ and acidic pH conditions.

## 1. Introduction

**Figure 1:**
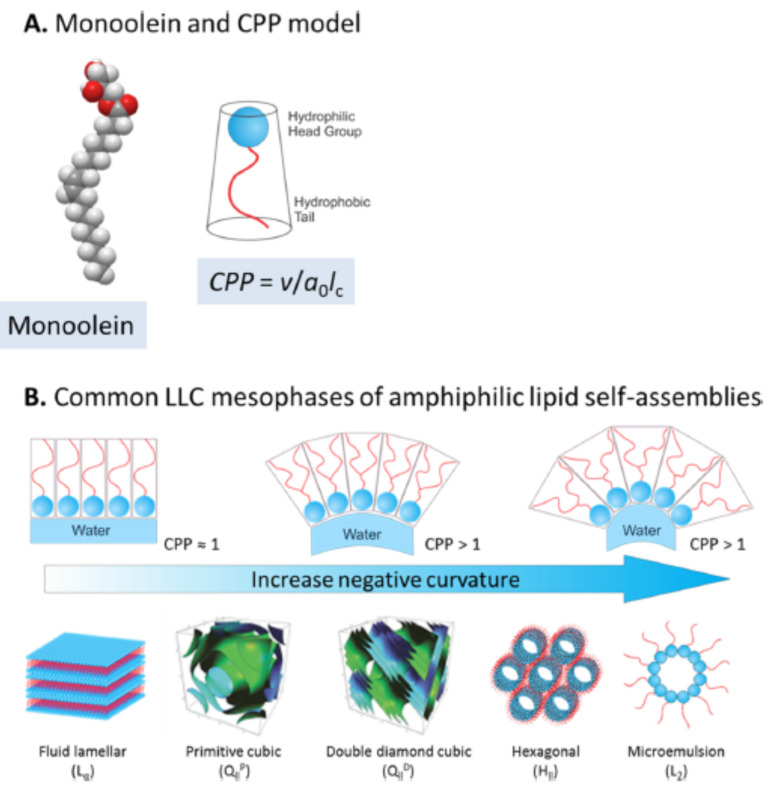
(**A**) Monoolein (MO) molecular model and critical packing parameter (CPP) representation, where *v* is the hydrophobic chain volume, *a_0_* is the effective headgroup area and *l_c_* is the effective hydrophobic chain length. (**B**) Structures of commonly observed self-assembled lyotropic liquid crystalline (LLC) mesophases of amphiphilic lipids such as MO, presented in the order of increasing negative lipid membrane curvature. Reproduced from Zhai et al. [6]. Copyright 2019, American Chemical Society.

Lipid-based cubosomes and hexosomes are a class of lipid nanoparticles containing the intriguing non-lamellar lyotropic liquid crystalline (LLC) mesophases, i.e., the inverse bicontinuous cubic (Q_II_) phase and the inverse hexagonal (H_II_) phase, respectively, which are formed by amphiphilic lipid self-assembly in aqueous conditions (Figure 1) [1,2,3,4,5,6]. Over the past three decades, the unique multidimensional and porous structural characteristics [7,8,9] of the non-lamellar LLC mesophases within cubosomes and hexosomes have driven a considerable amount of interest in a range of biomedical applications, including drug delivery [10,11,12,13,14,15,16,17] theranostic application [18] and imaging [19,20,21,22]. The Q_II_ phase inside cubosomes can be described as a continuous, tortuous lipid bilayer draped over an infinite periodic minimal surface and composed of two interpenetrating water channels, possessing a large interfacial area [23]. The H_II_ phase inside hexosomes is characterized by elongated water tubes lined by lipid layers and packed in a hexagonal array [24]. A large body of literature has demonstrated the advantages of non-lamellar LLC lipid nanoparticles as drug delivery systems, including the versatility of encapsulating hydrophilic and hydrophobic drugs with high encapsulation efficiency [25], ability to protect and deliver large biomolecules (proteins, peptides, DNAs) [15,26,27,28,29] and tunability and responsiveness to external stimuli for controlling drug release [30,31,32,33]. Furthermore, improved efficacy of the encapsulated drugs within cubosomes and hexosomes has been frequently demonstrated in in vivo preclinical models [34,35,36,37].

Responsiveness to external stimuli is often a desirable attribute of nanoparticle drug delivery systems as the goal is to deliver the drug at specific target sites at a controlled rate. In this regard, the tunability of non-lamellar LLC mesophases, i.e., the phase transition between the lamellar (Lα), Q_II_, H_II_ and the inverse micellar (L_2_) phase within the nanoparticles, in response to environmental factors such as temperature and pH, has become an active research area [22,30,31,33,38,39,40,41]. Different internal mesophases within nanoparticles can influence drug encapsulation efficiency and release rate [6,25,42], cytotoxicity profile [43] and in vivo biodistribution and efficacy [19]. For example, the responsiveness of the internal mesophase to pH has been achieved by the addition of pH-sensitive amphiphilic molecule such as a long-chain fatty acid or an ionizable lipid to the parent lipid system [30,44,45,46,47]. The mechanism of the pH-induced phase transition can be rationalized by the critical packing parameter (CPP) concept (CPP = *v/a_0_l_c_*), where *v* is the volume of the hydrocarbon chain, $a_{0}$ is the effective headgroup area and $l_{c}$ is the maximum length of the hydrocarbon chain (Figure 1) [48]. Our recent study demonstrated pH responsiveness of the internal mesophase of MO nanoparticles enriched by synthetic ionizable aminolipids with the amino headgroup possessing a pK_a_ around pH 7 [47]. The ionization state of the aminolipid population can be manipulated by varying the pH level above or below the apparent pK_a_ of the ionizable moiety at the lipid-water interface. Under acidic pH conditions, the aminolipid headgroup becomes positively charged and electrostatic repulsion between the headgroups can significantly enlarge the effective headgroup area, reducing the CPP value and causing a phase transition to a mesophase with lower negative interfacial curvature. Specifically, MO nanoparticles enriched by the aminolipids underwent a phase transition from the H_II_ to the Q_II_ mesophase as the pH was reduced [47].

Most of the current cubosome formulations utilize commercially available poly(ethylene oxide)–poly(propylene oxide) block copolymer (e.g., Pluronic F127 or F108), which do not have intrinsic responsiveness to physiologically relevant stimuli such as pH and temperature. As discussed above, responsiveness can be achieved by adding additional amphiphilic molecules; however, such formulations still require the presence of steric stabilizers to produce stable nanoparticulate dispersions. There have been a number of studies on alternative polymers to substitute for Pluronic polymers for preparing LLC lipid nanoparticles, and a range of synthetic and commercially available polymers [49], including amphiphilic brush copolymers [50,51], poly(ethylene glycol) (PEG)-conjugated lipids [51,52,53] and amphiphilic proteins [54], have been explored. Numerous studies have shown that the choice of steric stabilizer not only can influence the particle size, size distribution and the internal mesophase of the formed lipid nanoparticles [49,55], but can have benefits of reducing cytotoxicity profile [56,57] and manipulating complement response [52].

However, there has been very limited studies using responsive polymers to make non-lamellar lipid nanoparticles which exhibit phase transitions in response to environmental conditions. Recently, Chountoulesi et al. synthesized a stimuli-responsive polycationic block copolymer, poly(2-(dimethylamino)ethyl methacrylate)-b-poly(lauryl methacrylate) (PDMAEMA-b-PLMA), to stabilize for MO nanoparticles and indicated that the nanoparticles responded to pH and temperature changes [58]. However, the study did not directly identify the internal LLC mesophase but used the fractal dimension parameter as an indicator to suggest mesophase or morphological change. Another recent study by Jenni et al. conjugated the diketopyrrolopyrrole-porphyrin based photosensitizer to Pluronic F108 and used the conjugate to stabilize cubosomes [16]. Docetaxel as a model drug was loaded into the cubosomes stabilized by the photosensitizer-polymer conjugate and the drug release and efficacy was demonstrated to be enhanced following irradiation.

**Figure 2:**
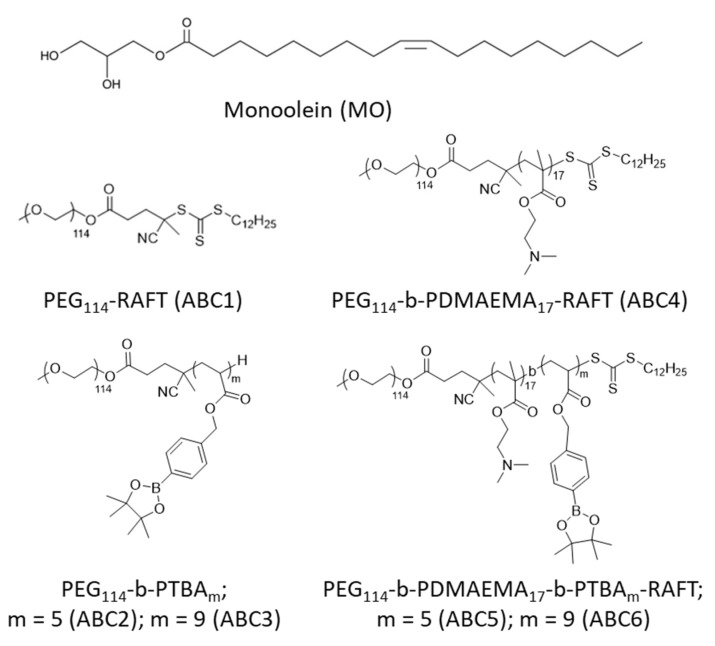
Chemical structures of MO and the synthetic ABCs used in the study.

Herein, we report the Reversible Addition-Fragmentation chain Transfer (RAFT) synthesis of a class of novel amphiphilic block copolymers (ABCs) which are dually functional. These ABCs not only act as a stabilizing agent for non-lamellar lipid nanoparticulate dispersions but also possess responsive chemical groups to impart an ability for LLC mesophase transition to occur with changes to physiologically and pathologically relevant pH, temperature and hydrogen peroxide (H_2_O_2_) conditions, such as in the gastrointestinal tract or in a tumor microenvironment (TME). The synthetic ABCs were designed with a hydrophobic segment to promote partitioning into the MO lipid layer, an extended hydrophilic PEG segment for steric stabilization, and responsive functional moieties (chemical structures given in Figure 2). The functional moiety of pinacol boronic ester in poly(4-(4,4,5,5-tetramethyl-1,3,2-dioxaborolan-2-yl)benzyl acrylate) (PTBA) is responsive to H_2_O_2_ [59], while the PDMAEMA is a dually responsive polymer in response to a change of pH and temperature [60]. MO was chosen because it is the most studied and well-characterized lipid in the formulation of non-lamellar lipid nanoparticles [6,61]. We utilized high-throughput formulation methodology to prepare MO nanoparticles stabilized by the synthetic ABCs at six different concentrations [62]. Direct identification of the LLC mesophase within the nanoparticles was undertaken by utilizing synchrotron small angle X-ray scattering (SAXS), and the effect of the ABC structure, concentration and three environmental factors (pH, temperature and H_2_O_2_) on the internal LLC mesophase of the formed particles was systematically investigated. This study reports the synthesis of novel, dually functional ABCs which can successfully stabilize MO nanoparticles and provide mesophase responsiveness to environmental factors; thereby further advancing the field of stimuli-responsive non-lamellar LLC lipid nanoparticles as prospective drug carriers.

## 2. Results

### 2.1. Synthesis of Stimuli-Responsive ABCs

To synthesize the stimuli-responsive ABCs, PEG methyl ether (PEG_114_, average *M*_n_ = 5000 g/mol) was firstly coupled with RAFT agent 4-cyano-4 (((dodecylthio) carbonothioyl)thio) pentanoic acid (CDPA) through Steglich esterification. CDPA is a commercially available RAFT agent that was widely used to control the polymerization of different types of monomers, including styrene, (meth)acrylate and (meth)acrylamide [63,64,65,66]. The long alkyl chain of CDPA allows it to work as the hydrophobic tail in ABC1. Next, ABC1 was chain extended with monomers (TBA and DMAEMA) at different ratios to afford ABC2-ABC6. Notably, the RAFT end-groups in ABC2 and ABC3 were removed via radical-induced reduction to compare the effect of different hydrophobic blocks on the formation of LLC mesophases. These polymers are well-controlled and exhibit low dispersities (*Ð* = 1.08–1.17). The detailed synthesis procedures are provided in the experimental section. The ^1^H nuclear magnetic resonance (NMR) spectra and gel permeation chromatography (GPC) curves are listed in Supplementary Information Figures S1–S12.

### 2.2. Formulation and Characterization of MO Nanoparticles Stabilized by the Synthetic ABCs

To assess the ability of the synthetic ABCs to make LLC mesophase-containing MO nanoparticles, the polymers were added at a concentration range of 0.5–3.0 mol% to a fixed amount of MO (20 mg/mL), followed by high power sonication. Initial visual examination showed that almost all six ABCs could disperse the lipid into milky nanoparticle solutions without visible lipid aggregates, but the samples became more translucent as the polymer concentration increased (Supplementary Information Table S1). The PEG_114_-*b*-PTBA_5_ polymer (ABC2) could not disperse the lipid at the lowest concentration (0.5 mol%) into a homogenous solution with a population of small lipid aggregates observed in the sample. The particle size and the size distribution of the formed nanoparticles were examined within 24 h of formulation, and results are given in Supplementary Information Table S2. The results were consistent with the morphological observation. The sample stabilized by 0.5 mol% ABC2 had the largest average particle size (301 nm) and was the most polydisperse (polydispersity index (PDI) = 0.38). The particle size of the majority of the MO nanoparticles was in the range of 140–300 nm and the PDI was in the range of 0.1–0.3, both within the expected range compared to previous studies of a wide range of LLC lipid nanoparticles [47,67].

**Figure 3:**
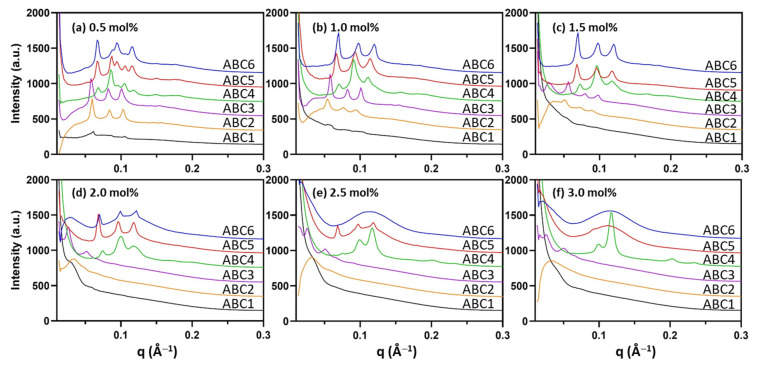
SAXS diffraction patterns of MO nanoparticles stabilized by the synthetic ABCs at (**a**) 0.5 mol%; (**b**) 1.0 mol%; (**c**) 1.5 mol%; (**d**) 2.0 mol%; (**e**) 2.5 mol%; (**f**) 3.0 mol% to the amount of MO. All measurements were performed at 25 °C.

The internal LLC mesophase of the formed nanoparticles was examined by high-throughput synchrotron SAXS and the one-dimensional SAXS profile of each formulation is given in Figure 3. The assigned LLC mesophase and the calculated lattice parameter (*a*) for each formulation is summarized in Table 1. Note that mesophase assignment of representative samples using the characteristic peak spacing ratio for each mesophase is given in Supplementary Figure S13. The results show that the internal mesophase of the formed nanoparticle depends on the polymer structure as well as the added polymer concentration. MO nanoparticles stabilized by 0.5 mol% PEG_114_-RAFT (ABC1) with a hydrophobic C12 end and a hydrophilic PEG_114_ block lost the internal Q_II_ phase to a large extent, which was then lost completely at higher polymer concentrations, indicating strong partitioning of the ABC1 polymer into the lipid layer and disruption of the parent MO cubic membrane packing.

Polymers ABC2 and ABC3 do not possess the C12 RAFT end-group and instead have 5 and 9 repeating units of TBA groups, respectively. ABC2-stabilized MO nanoparticles contained the primitive Q_II_ (Q_II_^P^) phase with symmetry group *Im3m* up to 1.5 mol% and in comparison, nanoparticles stabilized by ABC3 retained the Q_II_^P^ phase even at the highest concentration (3 mol%). Examination of the lattice parameters (*a*) revealed an interesting trend that increasing the concentration of polymer ABC2 and ABC3 had a swelling effect on the Q_II_^P^ phase with the *a* of 3.0 mol% ABC3-stabilized cubosome having the largest value of 178 Å. The increase in *a* indicates swelling of the internal water channels within the mesophase. However, it should be noted that the diffraction signals of the highly swollen Q_II_^P^ phase were relatively weak. Overall, the results indicate that polymers ABC1, ABC2 and ABC3 can be incorporated into the MO lipid membrane and reduce the negative membrane curvature to various degrees; such effect is in the order of ABC1 > ABC2 > ABC3 and increases with the concentration of the polymer within the system.

Interestingly, the PDMAEMA-containing polymers (ABC4, ABC5, ABC6) had the opposite effect of increasing the negative membrane curvature. In the case of the PEG_114_-*b*-PDMAEMA_17_-RAFT polymer (ABC4), the MO nanoparticle stabilized by 0.5 mol% ABC4 contained mixed mesophases of the Q_II_^P^ phase and the double diamond Q_II_ (Q_II_^D^) phase with *a* of 127 Å and 101 Å, respectively. As the polymer concentration increased, the *a* values for each phase gradually decreased and at the highest concentrations (2.5 mol% and 3 mol%), the internal mesophase transformed to co-existing Q_II_^D^ and H_II_ phases, further confirming the increase in the negative membrane curvature. The sub-set of PEG_114_-*b*-PDMAEMA_17_-*b*-PTBA_m_-RAFT polymers (ABC5 and ABC6) both acted similarly with a mesophase transition from the Q_II_^P^ phase to the L_2_ phase as the polymer concentration increased. At the lowest concentration (0.5 mol%), both ABC5- and ABC6-stabilized MO nanoparticles exhibited mixed Q_II_^P^ (*a* = 131 Å) and Q_II_^D^ (*a* = 103 Å) phases. The mesophase transition concentration, however, differed for the two ABCs with ABC6 causing a complete transition to the L_2_ phase at 2.5 mol% and ABC5 at 3.0 mol%. These results also indicate that the negative curvature promoting effect is in the order of ABC6 > ABC5 >ABC4 and increases with the concentration of the polymer.

### 2.3. H_2_O_2_-Responsiveness of the Formulated Nanoparticles

**Figure 4:**
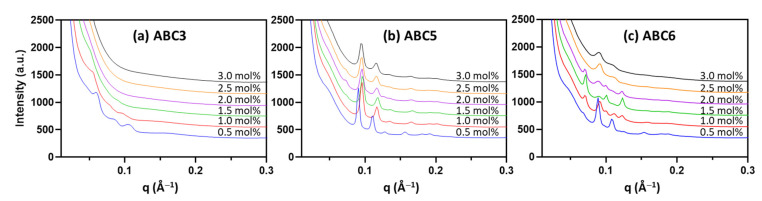
SAXS diffraction patterns of MO nanoparticles stabilized by PTBA-containing polymers, *viz.* ABC3 (**a**), ABC5 (**b**) and ABC6 (**c**) at various concentrations after incubation with 50 mM H_2_O_2_ solution. All measurements were performed at 25 °C.

The hydrophobicity of the PTBA-containing polymers is expected to decrease in a H_2_O_2_ environment due to H_2_O_2_-induced degradation of the pinacol boronic ester group followed by the loss of the aromatic ring [59]. Therefore, MO nanoparticles stabilized by PTBA-containing polymers may undergo LLC mesophase changes in the presence of H_2_O_2_, as the polymer side-group structure changes. To assess the mesophase responsiveness of the formulated nanoparticles stabilized by PTBA block-containing polymers, 50 mM H_2_O_2_ was added to the nanoparticulate dispersions stabilized by ABC3, ABC5 and ABC6 and the SAXS results are given in Figure 4 and Table 2. Polymer ABC4 with no PTBA block served as a control and results confirm no LLC mesophase changes in 50 mM H_2_O_2_ (Supplementary Information Figure S14).

Figure 4a and Table 2 show that after incubating the ABC3-stabilized MO nanoparticles with 50 mM H_2_O_2_ for one hour, only those stabilized by low polymer concentrations (0.5–1.0 mol%) still retained the Q_II_^P^ phase. However, nanoparticles stabilized by ABC3 at 1.5 mol% or higher concentrations lost the highly swollen Q_II_^P^ phase observed in the absence of H_2_O_2_ (Table 1), indicating that the PTBA block started degrading. However, after H_2_O_2_ incubation, ABC5-stabilized MO nanoparticles favored the formation of the Q_II_^D^ phase (Figure 4b; Table 2) instead of the Q_II_^P^ phase found under normal conditions (Figure 3; Table 1). In the case of the ABC6 samples, the Q_II_^D^ phase also appeared in the polymer concentration range of 2.5–3.0 mol% (Figure 4c).

The H_2_O_2_-induced mesophase changes of the MO nanoparticles stabilized by ABC5 and ABC6 were most distinct at high polymer concentrations. Under normal buffer conditions, ABC5 (3.0 mol%) and ABC6 (2.5–3.0 mol%) could stabilize MO nanoparticles containing the L_2_ phase (Figure 3; Table 1). As shown by Figure 4b,c, H_2_O_2_ incubation led to the transition from the L_2_ phase to the Q_II_^D^ phase. This mesophase transition clearly indicates a decrease of the negative membrane curvature, which can be attributed to the degradation of the hydrophobic PTBA group and less polymer insertion into the lipid matrix.

### 2.4. pH-Responsiveness of the Formulated Nanoparticles

PDMAEMA is a well-known dually-responsive functional block that changes water solubility in response to pH and temperature and therefore has been explored in many polymer-based drug delivery systems [60,68]. In the present study, the pH responsiveness of the LLC mesophase of the MO nanoparticles stabilized by ABC4, ABC5 and ABC6 (all containing the PDMAEMA block) was assessed at pH levels between 2 and 10. As a control, MO nanoparticles stabilized by ABC3 with no PDMAEMA block showed no mesophase changes with all formulations exhibiting the Q_II_^P^ phase in the pH range of 2–10 (Supplementary Information Figure S15).

**Figure 5:**
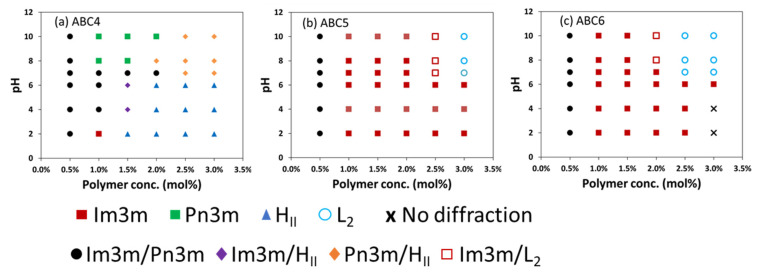
Mesophases of MO nanoparticles stabilized by PDMAEMA-containing polymers, *viz.* ABC4 (**a**), ABC5 (**b**) and ABC6 (**c**) at various concentrations in pH 2 to 10. All measurements were performed at 25 °C.

The pH-responsiveness of the MO nanoparticles stabilized by PDMAEMA-containing polymers depends on the polymer structure and concentration (Figure 5). For example, Figure 5a shows that 1.0 mol% ABC4 caused the internal LLC mesophase of the MO nanoparticles to transform from Q_II_^P^ (pH 2) to mixed Q_II_^P^/Q_II_^D^ (pH 4–7) to Q_II_^D^ (pH 8–10). At higher concentrations, the concentration effect of increasing the negative membrane curvature seemed to be the dominant effect as a large compositional space (2.0–3.0 mol%, pH 2–6) exhibited the H_II_ phase. It can also be observed that at 2.5–3 mol% ABC4, increasing pH caused a transition from H_II_ to mixed H_II_/Q_II_^D^ at neutral pH, which seemed to be in the opposite direction in terms of changing the membrane curvature compared to the 1.0 mol% sample. This could be due to the complexity of the apparent pK_a_ of the DMAEMA group which can depend on the polymer structure, the concentration in the system, the insertion into the membrane and the ionic strength of the buffer (further discussed in Section 3).

Due to the presence of the extra hydrophobic block (PTBA), ABC5 (Figure 5b) and ABC6 (Figure 5c) could stabilize MO nanoparticles that resisted pH-induced changes up to 2 mol% and 1.5 mol%, respectively. At higher concentrations, increasing the pH caused the Q_II_^P^ to L_2_ mesophase transformation at around neutral pH. A specific example is the sample stabilized by 2.5 mol% ABC6, which had the Q_II_^P^ phase at pH 2–6 and transited to the L_2_ phase at pH 7–10. Overall, these results indicate that pH-induced mesophase transitions in MO nanoparticles stabilized by the PDMAEMA-containing polymers occurs around neutral pH and increasing the pH leads to the formation of mesophases with higher negative membrane curvature.

### 2.5. Temperature-Responsiveness of the Formulated Nanoparticles

**Figure 6:**
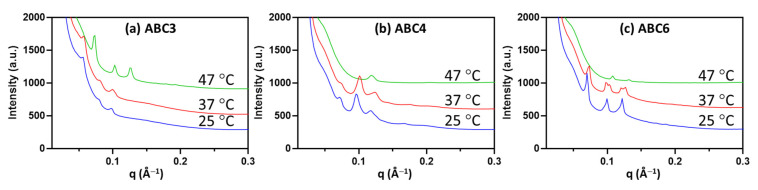
SAXS diffraction patterns of MO nanoparticles stabilized by 1.5 mol% ABC3 (**a**), 1.5 mol% ABC4 (**b**) and 1.5 mol% ABC6 (**c**) at neutral pH measured at three temperatures.

All previously described formulations (six polymers, six concentrations and seven buffer conditions) were screened by SAXS at three different temperatures, namely 25 °C, 37 °C and 47 °C (the highest sample environment temperature achievable at the synchrotron beamline), representing a total of 252 unique sample conditions. Due to the large number of data points, representative results are provided in Figure 6 to illustrate the effect of temperature on the mesophase behavior. In general, the mesophase behavior of the formulated nanoparticles with increasing temperature is as expected. Lipid self-assembly has intrinsic responsiveness to temperature as increasing temperature enhances hydrocarbon chain mobility and either reduces the lattice parameter of the unchanged mesophase or promotes a transition to a mesophase with higher interfacial negative curvature. As can be seen in the phase diagram of monoolein (MO) [69], a wedge-shaped molecule, increasing the temperature promotes the transition of MO-water systems from the Q_II_ phase to the H_II_ phase. Numerous studies have also shown such temperature effect in nanoparticulate form [45,54].

In this study, the temperature effect of increasing the negative membrane curvature is demonstrated by examples of MO nanoparticles stabilized by three polymers at 1.5 mol% under neutral pH condition (Figure 6). Specifically in the case of the ABC3-stabilized MO nanoparticles, the lattice parameter of the Q_II_^P^ phase reduced significantly from 157 Å at 25 °C and 37 °C to 121 Å at 47 °C, as can be seen from the diffraction peaks shifting to higher q values (Figure 6a). On the other hand, at 47 °C, ABC4-stabilized MO nanoparticles exhibited a cubic (mixed Q_II_^P^/Q_II_^D^) to H_II_ transition (Figure 6b), while ABC6-stabilized nanoparticles exhibited a Q_II_^P^ (*a* = 126 Å) to Q_II_^D^ (*a* = 83 Å) phase transition.

## 3. Discussion

The concept of CPP has been frequently used to describe the shape of an amphiphilic molecule and predict the effect of amphiphilic additives on the LLC mesophase of lipid-water systems formed under specific conditions [55,70]. While the CPP of small molecule additives can be calculated using some standard equations, CPP estimation for large polymers such as ABCs can be more complex. Multiple steps of calculation and experimental measurement are required to derive CPP estimates for polymers. For example, the polymer chain “pervade” volume is derived from the radius of gyration or root-mean-square end-to-end distance of the polymer chain. This is highly dependent on the solvent conditions. Experiments such as static light scattering and small-angle X-ray/neutron scattering are required to measure the radius of gyration. The polymer chain length can be estimated from the monomer units. However, it also depends on the folding/coiling of the chain. The effective area of the hydrophilic headgroup can also be measured experimentally or estimated theoretically. In general, the determination of CPP values for block copolymers is non-trivial. Nonetheless, the results reported herein, (Figure 1) show that the CPP is in the order of ABC6 > ABC5 > ABC4 > ABC3 > ABC2 > ABC1 under neutral PBS conditions. This trend is as expected because all six ABCs have the same hydrophilic block (PEG_114_ block), which presumably occupy the same headgroup area. ABC1 has the smallest CPP due to its small hydrophobic block (C_12_H_25_) and substitution of C_12_H_25_ to the PTBA_5-9_ block (ABC2 and ABC3) leads to increased CPP, attributable to the brush-like structures occupying larger volumes in the hydrophobic region. It is interesting to observe that adding a PDMAEMA_17_ group can dramatically increase the CPP and promote higher membrane curvature (for example, ABC4 versus ABC1). Even though PDMAEMA is largely considered as hydrophilic, our results indicate that it at least partially interacted with the MO lipid bilayer and led to a mesophase transformation to the H_II_ phase (ABC4) under neutral PBS conditions. This could be due to the increased backbone length. When both PTBA and PDMAEMA groups are present (ABC5 and ABC6), there is a transformation to the L_2_ phase which possesses the highest negative membrane curvature, further confirming strong incorporation of the backbone into the membrane layer. The exact mechanism of the polymer-lipid interaction is unknown and needs further investigation.

As a proof of concept, the responsiveness of the ABC-stabilized MO nanoparticles was examined by changing environmental factors such as H_2_O_2_, pH and temperature. The PTBA block is degradable in response to H_2_O_2_ and given the hydrophobic nature of the PTBA block, H_2_O_2_-induced degradation is expected to lead to LLC mesophase changes as the polymer becomes more hydrophilic and the inserted PTBA region is eliminated [59]. Indeed, when the MO nanoparticles stabilized by PTBA-containing polymers were incubated in 50 mM H_2_O_2_, a reduction of the membrane curvature indicated by the mesophase transformation from H_II_ or L_2_ phase to Q_II_ phase was observed (Table 1 vs. Table 2). High concentrations of H_2_O_2_ is one of the hallmarks of the TME and has been exploited to design smart H_2_O_2_-responsive materials to enhance targeted drug delivery and tumor treatment efficiency [71]. Therefore, the current study illustrates the prospect of designing responsive LLC nanoparticle drug delivery systems. Transformation of the H_II_ or L_2_ phase with slow drug release to the Q_II_ phase with high drug release rate [6] is desirable for systemic delivery where a burst release of drugs is only wanted at the tumour site.

The PDMAEMA moiety is pH-sensitive and has a reported pK_a_ around 7.3–7.5 in water [72]. The apparent pK_a_ at the lipid-water interface will be different [73]. At low pH, the moiety is protonated and the electrostatic repulsion between the surface charge groups should increase the occupied volume; at high pH, the moiety is deprotonated and becomes more aggregated [72]. The change of the LLC mesophase will then largely depend on the location of the dimethylamino group in the MO lipid bilayer. In the case of ABC5 and ABC6 (2–3 mol%), increasing the pH led to a Q_II_ (pH 2–6) to L_2_ transformation at around pH 7, indicating that deprotonation of the dimethylamino group at higher pH reduces effective headgroup area and increases the membrane curvature. Such transformation behavior can offer advantages in enhancing drug release from the Q_II_ phase in the slightly acidic TME [74] or enhancing the escape of the drug from the acidic endosomes/lysosomes [75]. While the current study needs optimization and further investigation of the lipid-polymer interaction, the value of the work lies in the successful demonstration of dually functional synthetic ABCs that can substitute for the commercial Pluronic polymer to stabilize non-lamellar LLC lipid nanoparticles, and impart responsiveness to physiologically and pathologically relevant H_2_O_2_, pH and temperature for controlled drug release.

## 4. Materials and Methods

### 4.1. Materials

MO was obtained from Nu-chek-Prep, Inc (Elysian, MN, USA) with purity >99% according to the manufacturer’s certificate. Phosphate buffer saline, ethanol and H_2_O_2_ solution (30% *w/w* in H_2_O) were purchased from Sigma-Aldrich (Bayswater, Australia). Milli-Q water (18.2 MΩ.cm) obtained from a Milli-Q Direct water purification system (Merck, Bayswater, Australia) was used for all sample preparations.

RAFT agent 4-cyano-4 (((dodecylthio)carbonothioyl)thio) pentanoic acid (CDPA; 97%) was purchased from Boron Molecular (Noble Park, Australia). Poly(ethylene glycol) methyl ether (PEG_114_, average *M*_n_ = 5000 g/mol), 2-(dimethylamino)ethyl methacrylate (DMAEMA) and 1-methyl-1,4-cyclohexadiene were purchased from Sigma-Aldrich (Bayswater, Australia). 2,2′-Azobis(isobutyronitrile) (AIBN) and benzoyl peroxide were purchased from Wako Pure Chemical Industries, Ltd. The monomer 4-(4,4,5,5-tetramethyl-1,3,2-dioxaborolan-2-yl)benzyl acrylate (TBA) was synthesized according to a procedure reported previously [59]. All other solvents were obtained from commercial sources and were used as received unless noted otherwise.

^1^H Nuclear magnetic resonance spectra (NMR) were recorded on a Bruker Avance 400 NMR spectrometer at frequencies of 400 MHz. NMR chemical shifts (δ) are reported in ppm and were calibrated against residual solvent signal of CDCl_3_ (δ 7.26). Samples were dissolved in CDCl_3_ at 5–10 mg mL^−1^. The data are reported as chemical shift (δ).

Gel permeation chromatography (GPC) was performed on a system comprising a Shimadzu LC-20AT pump, Shimadzu RID-20A refractive index detector, and SPD-20A UV−Visible detector. The GPC is equipped with a guard column (WAT054415) and 3×Waters GPC columns (WAT044238, WAT044226, WAT044235, 300 mm × 7.8 mm). The eluent is DMF with 10 mM LiBr and eluted at 1 mL/min for 45 min in total. The columns were kept at 40 °C. The samples were dissolved in DMF with 10 mM LiBr, filtered through 0.20 μm syringe filters. A calibration curve was obtained from poly(methyl methacrylate) (PMMA) standards (Agilent, Mulgrave, Australia) ranging from 960 to 1,568,000 g mol^−1^.

### 4.2. Synthesis and Characterization of ABC1 (PEG_114_-RAFT)

The ABC1 (PEG_114_-RAFT) was synthesized via a procedure reported previously [63]. The sample was analyzed by ^1^H NMR and GPC. ^1^H NMR spectrum (400 MHz, CDCl_3_): δ 4.25 (t, 2H), 3.45–3.81 (m, 412H), 3.36 (s, 3H), 3.31 (t, 2H), 2.37–2.65 (m, 4H), 1.86 (s, 3H), 1.69 (m, 2H), 1.25–1.38 (b, 18H), 0.86 (t, 3H). GPC (DMF, PMMA standards): *M*_n_ = 12,300 g/mol, *Ð* = 1.08.

### 4.3. Synthesis and Characterization of ABC2 (PEG_114_-PTBA_5_)

PEG_114_-RAFT (300.0 mg, 55.6 μmol, 1.0 equiv.), TBA (96.1 mg, 333.6 μmol, 6.0 equiv.) and AIBN (1.8 mg, 11.1 μmol, 0.2 equiv.) were dissolved in 1.2 mL 1,4-dioxane and transferred to a Schlenk flask. The oxygen inside the flask was removed by 3 cycles of freeze-pump-thaw and refilled with argon in the third cycle. The reaction was stopped by cooling to room temperature after being immersed in a 65 °C oil bath for 20 h. The synthesized PEG_114_-PTBA_5_-RAFT was purified by precipitation in hexane and dried under reduced pressure. To remove the RAFT end-group, the PEG_114_-PTBA_5_-RAFT, benzoyl peroxide (35.4 mg, 146.0 μmol) and 1-methyl-1,4-cyclohexadiene (68.6 mg, 730.0 μmol) were dissolved in 1.0 mL *N,N*-dimethylformamide and transferred to a Schlenk flask. The oxygen inside the flask was removed by 3 cycles of freeze-pump-thaw and refilled with argon in the third cycle. The reaction was stopped by cooling to room temperature after being immersed in a 100 °C oil bath for 4 h. The polymer PEG_114_-PTBA_5_ was purified by precipitation in hexane and dried under reduced pressure. ^1^H NMR spectrum (400 MHz, CDCl_3_): δ 7.77 (b, 9H), 7.32 (b, 9H), 5.06 (b, 10H), 3.53–3.77 (b, 457H), 3.38 (s, 3H), 1.60–2.53 (b, 40H), 1.33 (b, 50H). GPC (DMF, PMMA standards): *M*_n_ = 13,500 g/mol, *Ð* = 1.12.

### 4.4. Synthesis and Characterization of ABC3 (PEG_114_-PTBA_9_)

ABC3 was synthesized in a similar procedure as ABC2. ^1^H NMR spectrum (400 MHz, CDCl_3_): δ 7.76 (b, 16H), 7.32 (b, 18H), 4.99 (b, 19H), 3.53–3.77 (b, 455H), 3.38 (s, 3H), 1.60–2.53 (b, 27H), 1.31 (b, 86H). GPC (DMF, PMMA standards): *M*_n_ = 13,900 g/mol, *Ð* = 1.14.

### 4.5. Synthesis and Characterization of ABC4 (PEG_114_-PDMAEMA_17_-RAFT)

PEG_114_-RAFT (1.0 g, 185.0 μmol, 1.0 equiv.), DMAEMA (0.58 g, 3.7 mmol, 20.0 equiv.) and AIBN (6.1 mg, 37.0 μmol, 0.2 equiv.) were dissolved in 3.0 mL 1,4-dioxane and transferred to a Schlenk flask. The oxygen inside the flask was removed by 3 cycles of freeze-pump-thaw and refilled with argon in the third cycle. The reaction was stopped by cooling to room temperature after being immersed in a 60 °C oil bath for 24 h. The synthesized PEG_114_-PDMAEMA_17_-RAFT was purified by precipitation in hexane and dried under reduced pressure. ^1^H NMR (400 MHz, CDCl_3_): δ 3.99–4.16 (b, 34H), 3.55–3.75 (b, 456H), 3.37 (s, 3H), 2.52–2.71 (b, 39H), 2.18–2.44 (b, 112H), 1.57–2.04 (b, 39H), 1.18–1.37 (b, 24H), 0.82–1.15 (b, 51H). GPC (DMF, PMMA standards): *M*_n_ = 12,700 g/mol, *Đ* = 1.17.

### 4.6. Synthesis and Characterization of ABC5 (PEG_114_-PDMAEMA_17_-PTBA_5_-RAFT)

PEG_114_-PDMAEMA_17_-RAFT (300.0 mg, 36.0 μmol, 1.0 equiv.), TBA (63.0 mg, 216.0 μmol, 6.0 equiv.) and AIBN (2.4 mg, 14.4 μmol, 0.4 equiv.) were dissolved in 1.0 mL 1,4-dioxane and transferred to a Schlenk flask. The oxygen inside the flask was removed by 3 cycles of freeze-pump-thaw and refilled with argon in the third cycle. The reaction was stopped by cooling to room temperature after being immersed in a 65 °C oil bath for 24 h. The synthesized PEG_114_-PDMAEMA_17_-PTBA_5_-RAFT was purified by precipitation in hexane and dried under reduced pressure. ^1^H NMR (400 MHz, CDCl_3_): δ 7.69–7.82 (b, 8H), 7.26–7.36 (b, 11H), 4.81–5.16 (b, 9H), 3.99–4.27 (b, 34H), 3.55–3.75 (b, 401H), 3.37 (s, 3H), 2.55–2.75 (b, 30H), 2.16–2.52 (b, 100H), 1.65–2.04 (b, 35H), 1.18–1.39 (b, 59H), 0.82–1.15 (b, 47H). GPC (DMF, PMMA standards): *M*_n_ = 14,500 g/mol, *Đ* = 1.13.

### 4.7. Synthesis and Characterization of ABC6 (PEG_114_-PDMAEMA_17_-PTBA_9_-RAFT)

ABC6 was synthesized in a similar procedure as ABC5. ^1^H NMR (400 MHz, CDCl_3_): δ 7.69–7.83 (b, 16H), 7.26–7.36 (b, 13H), 4.81–5.16 (b, 19H), 3.99–4.27 (b, 38H), 3.55–3.75 (b, 456H), 3.37 (s, 3H), 2.23–3.16 (b, 189H), 1.65–2.04 (b, 38H), 1.18–1.39 (b, 99H), 0.82–1.15 (b, 56H). GPC (DMF, PMMA standards): *M*_n_ = 15,300 g/mol, *Đ* = 1.14.

### 4.8. Formulation of ABC-Stabilized Nanoparticles

ABC-stabilized MO-based nanoparticles were prepared by adding ABC aqueous solutions to MO dry film, followed by sonication. Each formulation contained 20 mg of MO, which was first dissolved in ethanol and then evaporated overnight using a vacuum oven at 40 °C to obtain the dry lipid film. ABCs were solubilized in PBS buffer and equilibrated at room temperature for at least 48 h. ABC solutions were added to MO at 0.5%, 1.0%, 1.5%, 2.0%, 2.5% and 3.0% (mol/mol) to MO. The final sample volume was kept at 0.5 mL. Samples were then sonicated using a probe sonicator (Qsonica, Newtown, CT, USA) at a frequency of 30 kHz, with a 5 s on, 5 s off mode for a total sonication time of 2 min. Freshly prepared samples were examined visually and their dispersibility and appearance were recorded (Table S1).

To examine the H_2_O_2_ and pH responsiveness of the internal LLC mesophase of the formed nanoparticles, pre-made nanoparticles were diluted (1:1 ratio) with either 50 mM H_2_O_2_ solution, or PBS buffer with adjusted pH levels between 2 to 10 using hydrogen chloride or sodium hydroxide solution. The samples were incubated for one hour before SAXS examination.

To examine the temperature responsiveness, nanoparticle samples were mounted to the custom-designed plate holder at the SAXS/WAXS beamline at the Australian Synchrotron, and temperature was controlled in situ by a circulating water bath for scanning at 25 °C, 37 °C and 47 °C.

### 4.9. High Throughput Synchrotron SAXS Characterization

The SAXS experiment was performed at the SAXS/WAXS beamline at the Australian Synchrotron, part of ANSTO. The beamline used X-ray of wavelength λ = 1.033 Å (12.0 keV) with a typical flux of approximately 10^13^ photons/s. The sample to detector distance was chosen as 1.6 m which provided a *q*-range of 0.01–0.5 Å^−1^ (scattering vector *q* = 4*π* sin(*θ*)/*λ* where *θ* is the scattering angle and *λ* is the wavelength). Two-dimensional X-ray diffraction images were recorded on a Decris-Pilatus 1-M detector using an in-house IDL-based ScatterBrain software. The scattering images were integrated into one dimensional plots of intensity versus *q* for phase identification. A silver behenate standard (d = 58.38 Å) was used for calibration. The exposure time for each sample was 1 s. Prepared nanoparticles (100 µL) were loaded in UV-clear half-area 96-well microplate (Greiner Bio-One) and mounted to the high throughput sample-holder at the beamline.

### 4.10. SAXS Data Analysis

The one-dimensional SAXS data were analyzed using an IDL-based AXcess software package [76]. This program identifies LLC mesophases and calculates the lattice parameter. Phase identification was based on the relative distance of the Bragg peaks in the scattering profile, which corresponds to diffraction planes defined by their (*hkl*) Miller indices. Lattice parameter (*a*) was calculated using the equation *a* = *d*(*h*^2^+*k*^2^+*l*^2^)^1/2^ for cubic phase or *a* = *d*(*h*^2^+*k*^2^+*hk*)^1/2^ for hexagonal phase where *d* is the spacing between the diffraction planes, defined by Bragg’s law *d* = 2*π*/*q*.

## 5. Conclusions

In this study, a series of dual-functional ABCs was designed containing a hydrophobic part to partition into lipid layers, a hydrophilic part to exert steric stabilization for nanoparticle dispersions and a PTBA and/or PDMAEMA group that could respond to environmental factors. Using high throughput formulation and synchrotron SAXS techniques, we have successfully demonstrated that the synthetic ABCs could stabilize MO nanoparticles containing a range of LLC mesophases, which can respond to H_2_O_2_, pH and temperature. Notably, a mesophase transformation from the slow drug release H_II_/L_2_ phase to the high drug release Q_II_ phase can be induced in PTBA- or PDMAEMA-containing polymer-based nanoparticles under elevated H_2_O_2_ or acidic pH conditions, which are hallmarks of disease sites such as tumors. Future studies are planned to investigate model drug loading and release using these stimuli-responsive non-lamellar lipid nanoparticles, as well as cytotoxicity, cellular uptake and endosomal escape. The findings in this study may pave a new path to develop stimuli-responsive lipid nanoparticles that can promote drug release at specific target sites and enhance therapeutic efficacy.

## Figures and Tables

**Table 1:** Mesophase identification and calculated lattice parameters (Å) of the MO nanoparticles stabilized by the synthetic ABCs.

mol%	ABC1	ABC2	ABC3	ABC4	ABC5	ABC6
0.5	Weak Im3m signal	Im3m (145)	Im3m (153)	Pn3m (101); Im3m (127)	Im3m (131); Pn3m (103)	Im3m (130) Pn3m (103)
1.0	Weak Im3m signal	Im3m (162)	Im3m (153)	Pn3m (98); Im3m (115)	Im3m (132)	Im3m (126)
1.5	ND	Im3m (174)	Im3m (157)	Pn3m (92); Im3m (112)	Im3m (130)	Im3m (126)
2.0	ND	ND	Im3m (176)	Pn3m (89); Im3m (112)	Im3m (129)	Im3m (125)
2.5	ND	ND	Im3m (176)	Pn3m (90); H_2_ (61)	Im3m (130) L_2_	L_2_
3.0	ND	ND	Im3m (178)	Pn3m (90); H_2_ (62)	L_2_	L_2_

Note: Im3m refers to the primitive Q_II_ (Q_II_^P^) phase with the symmetry group *Im3m*; Pn3m refers to the double-diamond Q_II_ (Q_II_^D^) phase with the symmetry group *Pn3m*; H_II_ refers to the inverse hexagonal phase: L_2_ refers to the inverse micellar phase; ND refers to no distinctive diffraction pattern identified.

**Table 2:** Mesophase identification and calculated lattice parameters (Å) of the MO nanoparticles stabilized by the synthetic ABCs in the presence of 50 mM H_2_O_2_.

mol%	ABC3	ABC5	ABC6
0.5	Im3m (148)	Pn3m (98)	Pn3m (100)
1.0	Im3m (161)	Pn3m (92)	Im3m (122) Pn3m (97)
1.5	ND	Pn3m (91)	Im3m (125) Pn3m (100)
2.0	ND	Pn3m (93) Im3m (weak)	Im3m (120) Pn3m (100)
2.5	ND	Pn3m (94) (Im3m weak)	Pn3m (100)
3.0	ND	Pn3m (94)	Pn3m (101)

Note: Im3m refers to the primitive Q_II_ (Q_II_^P^) phase with symmetry group *Im3m*; Pn3m refers to the double-diamond Q_II_ (Q_II_^D^) phase with symmetry group *Pn3m*; ND refers to no distinctive diffraction pattern identified.

## Data Availability

The data presented in this study are available on request from the corresponding author.

## Supplementary Materials

The following are available online. Table S1: Visual examination of the nanoparticles stabilized by the synthetic polymers, Table S2: Particle size and size distribution of the nanoparticles measured by DLS, Figures S1–S12: ^1^H NMR and GPC data of the synthesized polymers, Figure S13: Representative mesophase identification, Figure S14: SAXS diffraction patterns of ABC4-stabilized nanoparticles under H_2_O_2_ condition, Figure S15: pH-composition partial phase diagram of ABC3-stabilized nanoparticles.

## Sample Availability

Samples of the compounds, monoolein and the polymers are available from the authors.
