# Supplementary material for: Novel Amphiphilic Block Copolymers for the Formation of Stimuli-Responsive Non-Lamellar Lipid Nanoparticles
Source: Molecules. 2021 Jun 15;26(12):3648. doi: 10.3390/molecules26123648 (PMC8232580; doi:10.3390/molecules26123648)
Supplement: Supplementary file 1 [file molecules-26-03648-s001.zip › molecules-1254760-supplementary.pdf]

Supplementary Information

Novel amphiphilic block copolymers for the formation of stimuli-responsive non-lamellar lipid nanoparticles

Jiali Zhai,[1*] Bo Fan,[2] San H. Thang,[2] Calum J. Drummond[1*]

[1]School of Science, STEM College, RMIT University, Melbourne, Vic 3000, Australia
[2]School of Chemistry, Monash University, Clayton, Vic 3800, Australia

Additional methods

Dynamic light scattering

Nanoparticle hydrodynamic diameter and polydispersity index (PDI) were measured using a Malvern Zetasizer plate reader based on a dynamic light scattering (DLS) method. Nanoparticles were diluted 20 times and measured in 96-well plates at 25 °C. Triplicate measurements with a minimum of 12 runs for each measurement were performed for each sample and the results were recorded in Table S2.

Table S1. Visual examination of the MO nanoparticles stabilized by the synthetic ABCs added at various concentrations.

mol%	ABC1	ABC2	ABC3	ABC4	ABC5	ABC6
0.5	Milky	Milky with aggregates	Milky	Milky	Milky	Milky
1.0	Milky	Milky	Milky	Milky	Milky	Milky
1.5	Translucent	Milky	Milky	Milky	Milky	Milky
2.0	Translucent	Milky	Milky	Translucent	Milky	Milky
2.5	Translucent	Milky	Milky	Translucent	Translucent	Milky
3.0	Translucent	Translucent	Translucent	Translucent	Translucent	Milky

Table S2. *Z*-average hydrodynamic size (nm) and polydispersity index (PDI) of the MO nanoparticles stabilized by the synthetic ABCs.

mol%	ABC1		ABC2		ABC3		ABC4		ABC5		ABC6	
	Size (nm)	PDI	Size (nm)	PDI	Size (nm)	PDI	Size (nm)	PDI	Size (nm)	PDI	Size (nm)	PDI
0.5	211±2	0.15±0.03	301±11	0.38±0.02	241±2	0.15±0.03	295±2	0.18±0.03	273±5	0.14±0.03	217±3	0.17±0.03
1.0	173±2	0.23±0.03	167±2	0.16±0.03	230±3	0.14±0.04	213±2	0.15±0.03	175±3	0.13±0.05	224±5	0.20±0.03
1.5	156±1	0.18±0.02	160±1	0.13±0.01	222±2	0.17±0.02	163±1	0.14±0.03	190±1	0.14±0.03	273±8	0.25±0.03
2.0	174±6	0.23±0.01	176±3	0.13±0.05	245±3	0.19±0.02	158±3	0.10±0.03	209±4	0.13±0.02	222±3	0.24±0.01
2.5	160±2	0.18±0.04	192±2	0.12±0.03	239±4	0.24±0.01	156±2	0.08±0.05	204±2	0.19±0.01	244±3	0.31±0.06
3.0	157±2	0.18±0.03	202±1	0.17±0.02	215±3	0.21±0.01	186±4	0.07±0.02	141±1	0.22±0.01	180±4	0.29±0.01

Note: Results are given as average ± standard deviation based on three independent measurements.

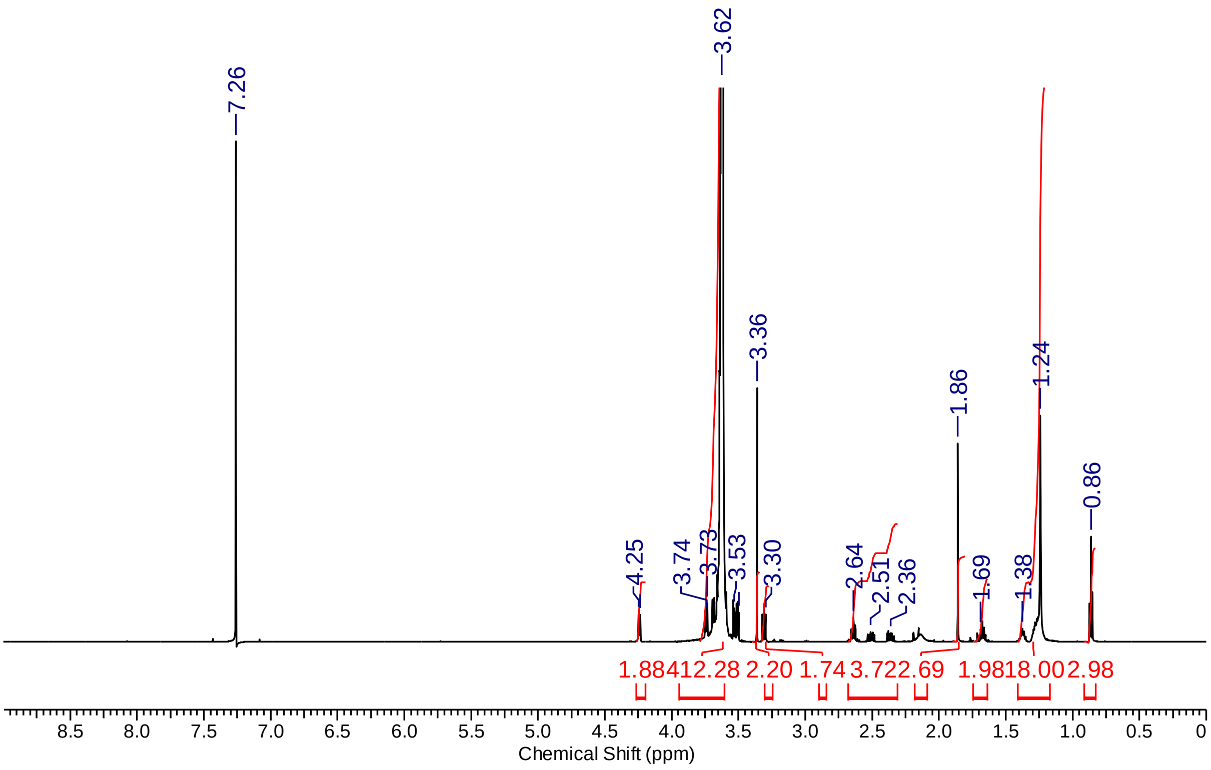

Figure S1. ^{1}H NMR spectrum (400 MHz, $CDCl_3$) of ABC1 (PEG_{114}-RAFT).

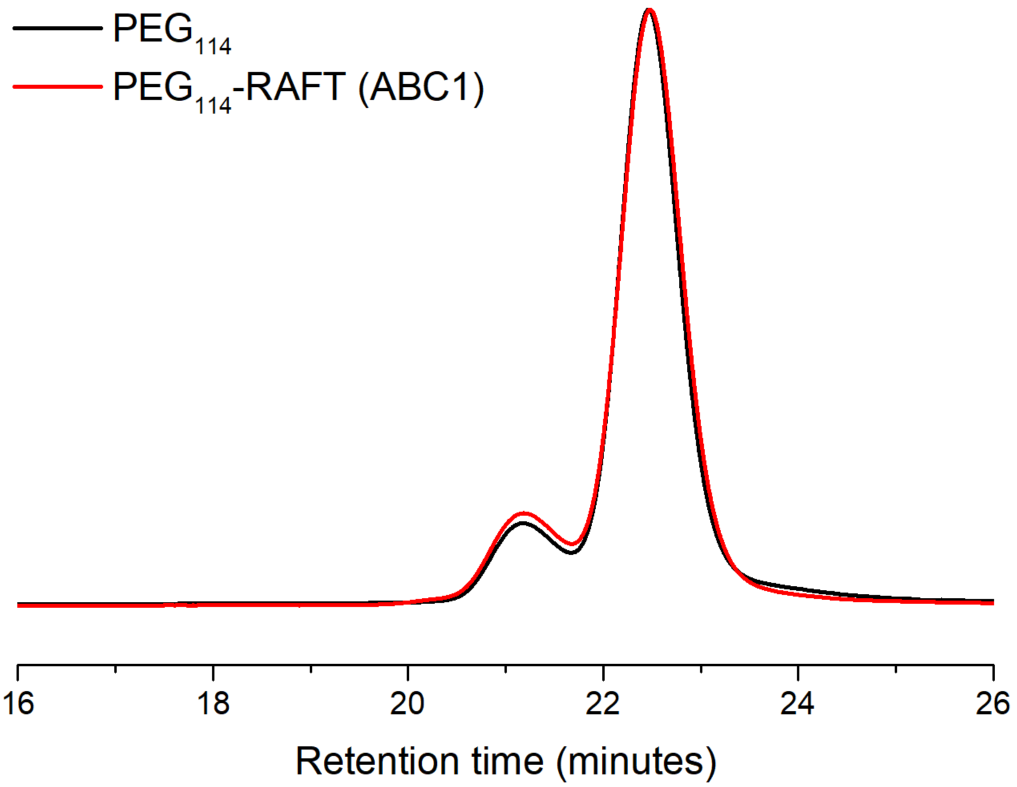

Figure S2. GPC traces of commercial PEG_{114} (M_n = 12,200 g/mol, $Đ$=1.08) and PEG_{114}-RAFT (ABC1, M_n = 12,300 g/mol, $Đ$ =1.08) (DMF GPC, PMMA standards). *Note: the shoulder peak comes from the small amount of PEG_{228} in the commercial source.*

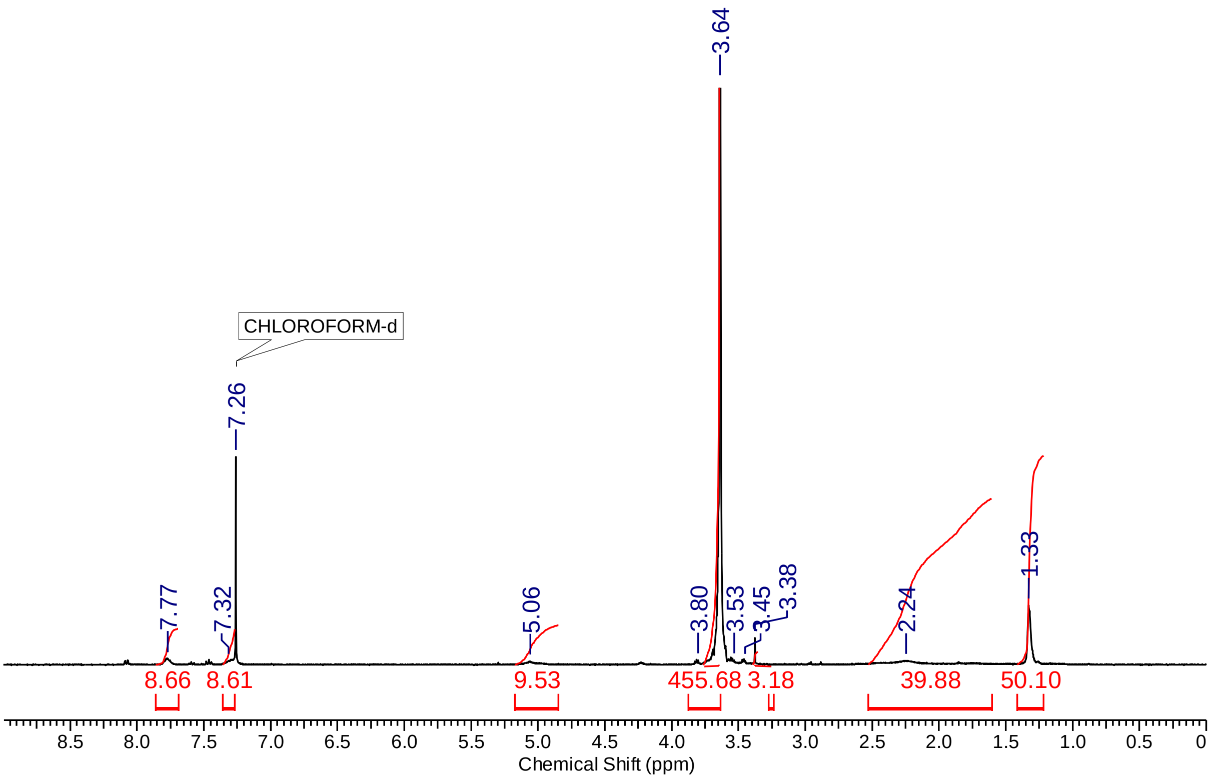

Figure S3. ^{1}H NMR spectrum (400 MHz, $CDCl_3$) of ABC2 (PEG_{114}-$PTBA_5$).

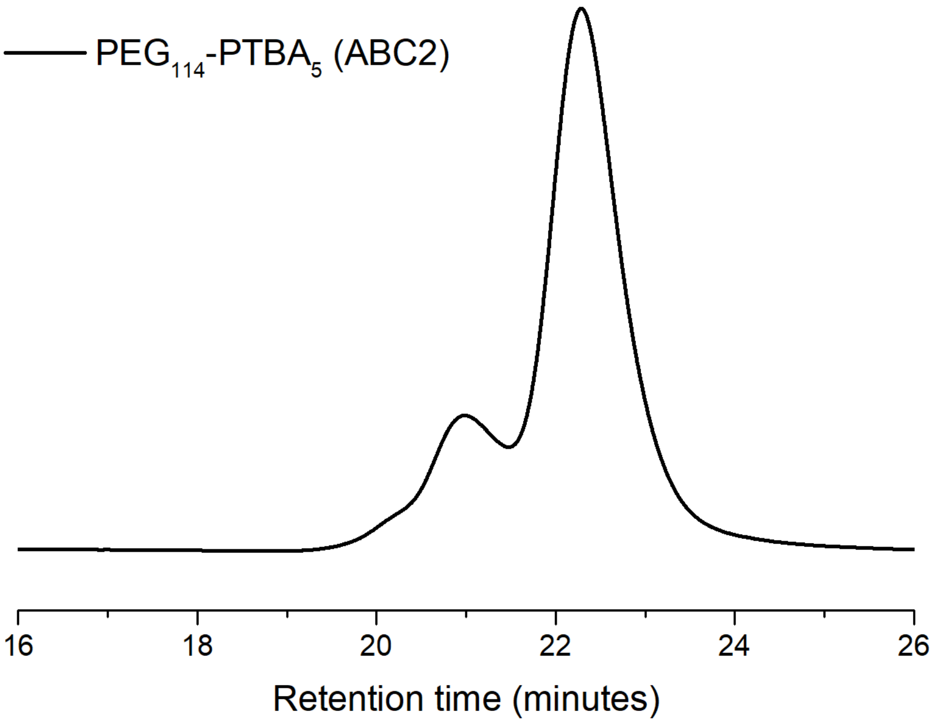

Figure S4. GPC trace of PEG_{114}-$PTBA_5$ (ABC2, M_n = 13,500 g/mol, $Đ$ =1.12) (DMF GPC, PMMA standards).

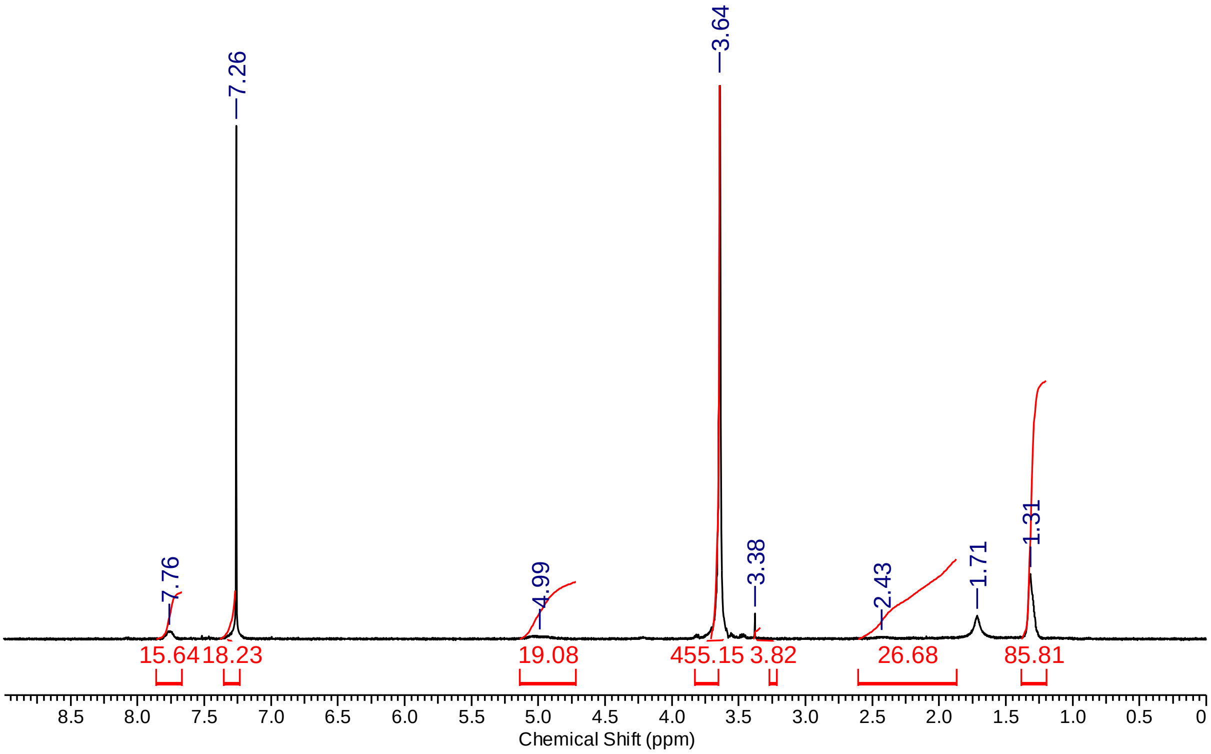

Figure S5. ^{1}H NMR spectrum (400 MHz, $CDCl_3$) of ABC3 (PEG_{114}-$PTBA_9$).

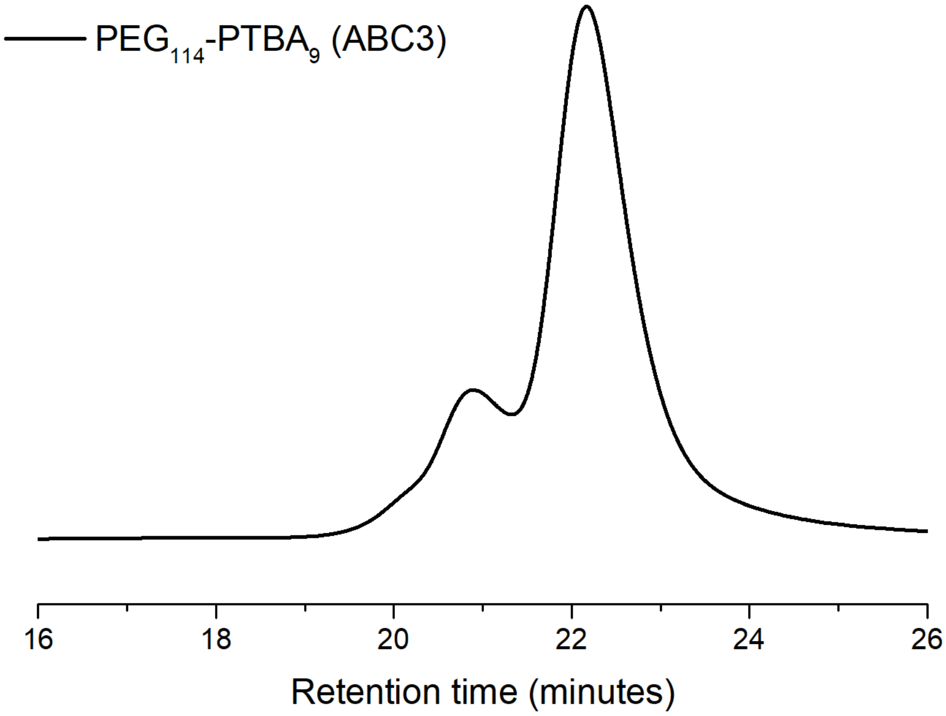

Figure S6. GPC trace of PEG_{114}-$PTBA_9$ (ABC3, M_n = 13,900 g/mol, $Đ$ =1.14) (DMF GPC, PMMA standards).

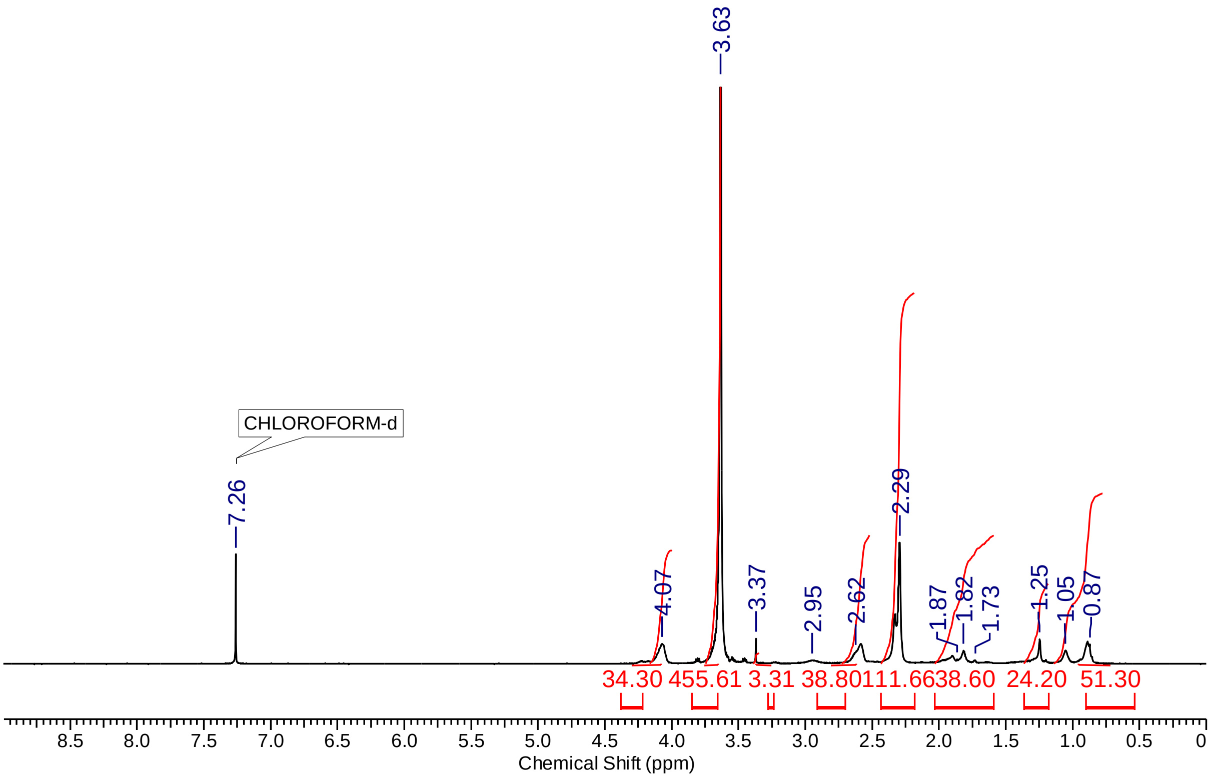

Figure S7. ^{1}H NMR spectrum (400 MHz, $CDCl_3$) of ABC4 (PEG_{114}-$PDMAEMA_{17}$-RAFT).

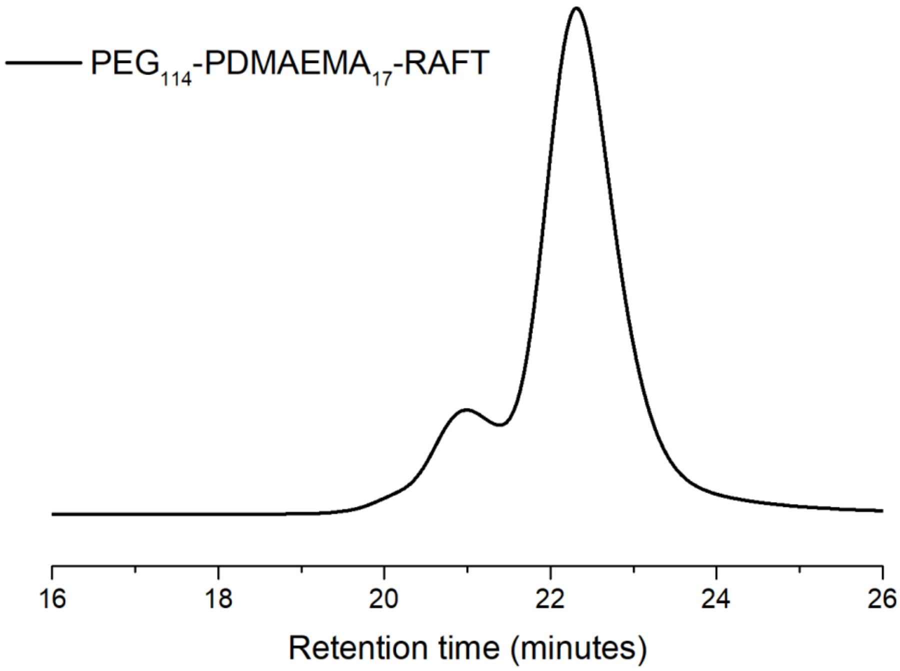

Figure S8. GPC trace of PEG_{114}-$PDMAEMA_{17}$-RAFT (ABC4, M_n = 12,700 g/mol, $Đ$ =1.17) (DMF GPC, PMMA standards).

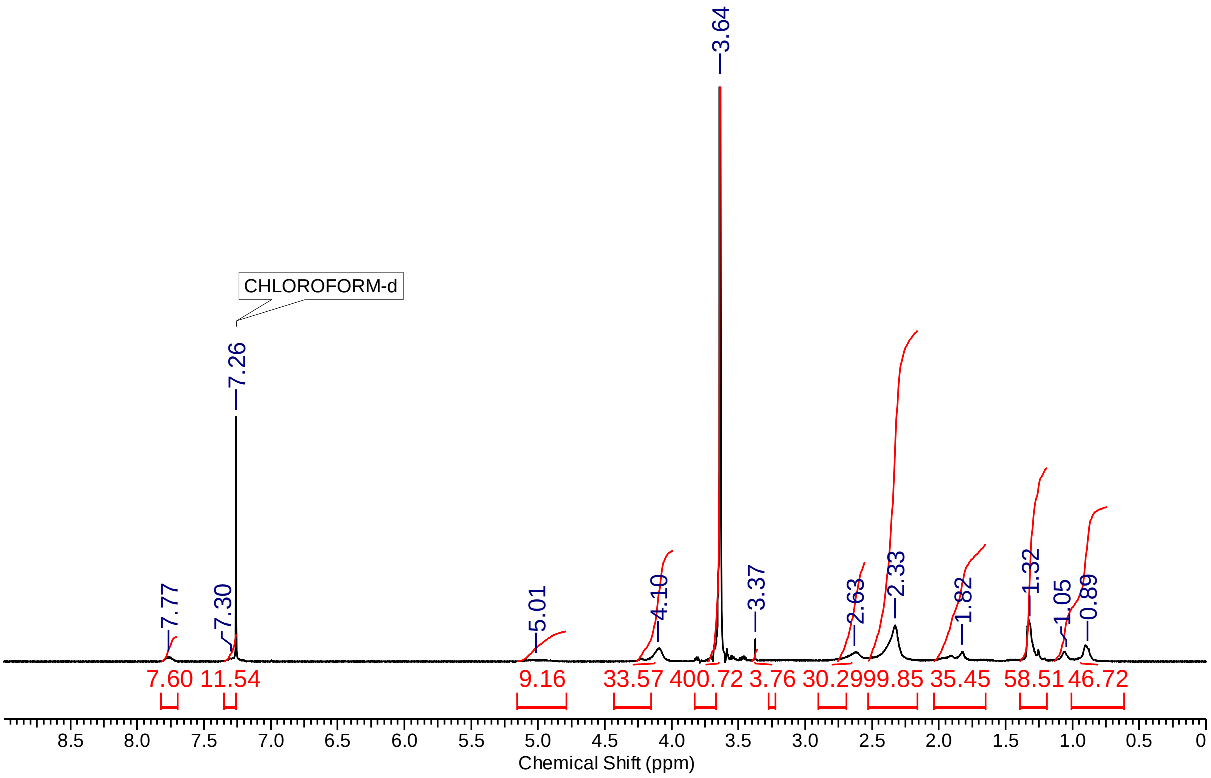

Figure S9. ^{1}H NMR spectrum (400 MHz, $CDCl_3$) of ABC5 (PEG_{114}-$PDMAEMA_{17}$-$PTBA_5$-RAFT).

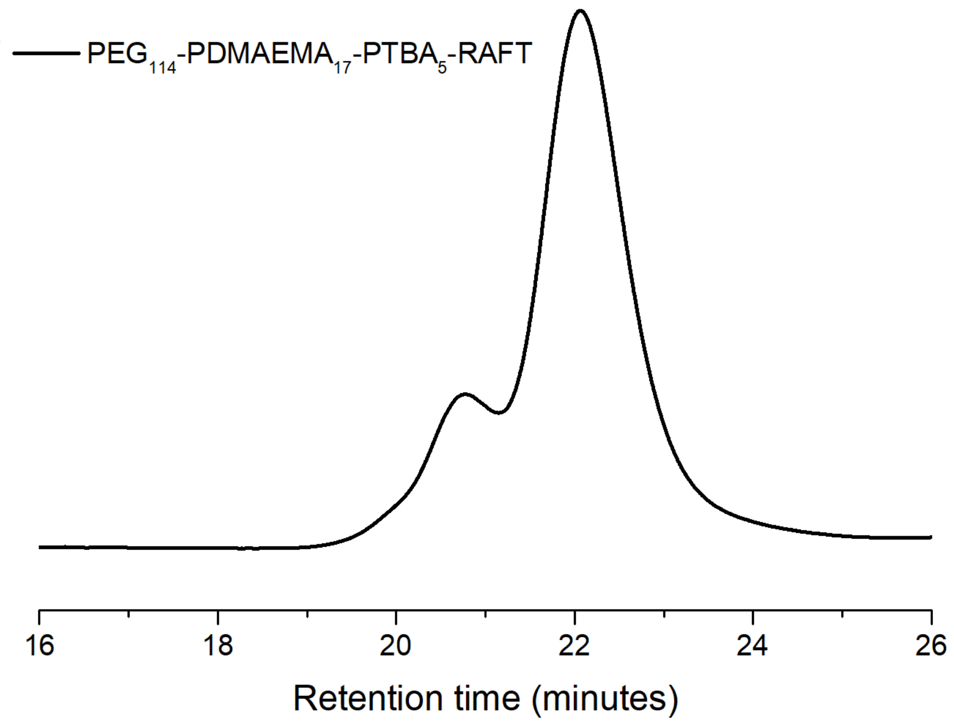

Figure S10. GPC trace of PEG_{114}-$PDMAEMA_{17}$-$PTBA_5$-RAFT (ABC5, M_n = 14,500 g/mol, $Đ$ =1.13) (DMF GPC, PMMA standards).

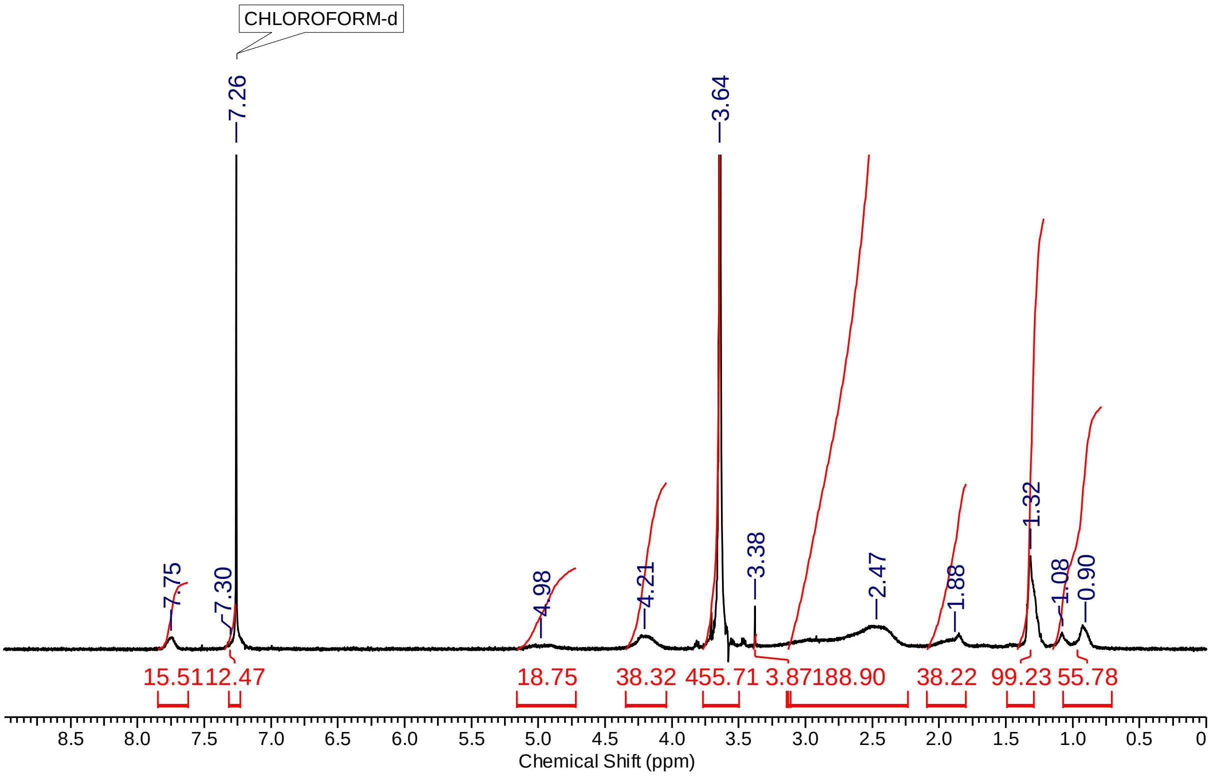

Figure S11. ^{1}H NMR spectrum (400 MHz, $CDCl_3$) of ABC6 (PEG_{114}-$PDMAEMA_{17}$-$PTBA_9$-RAFT).

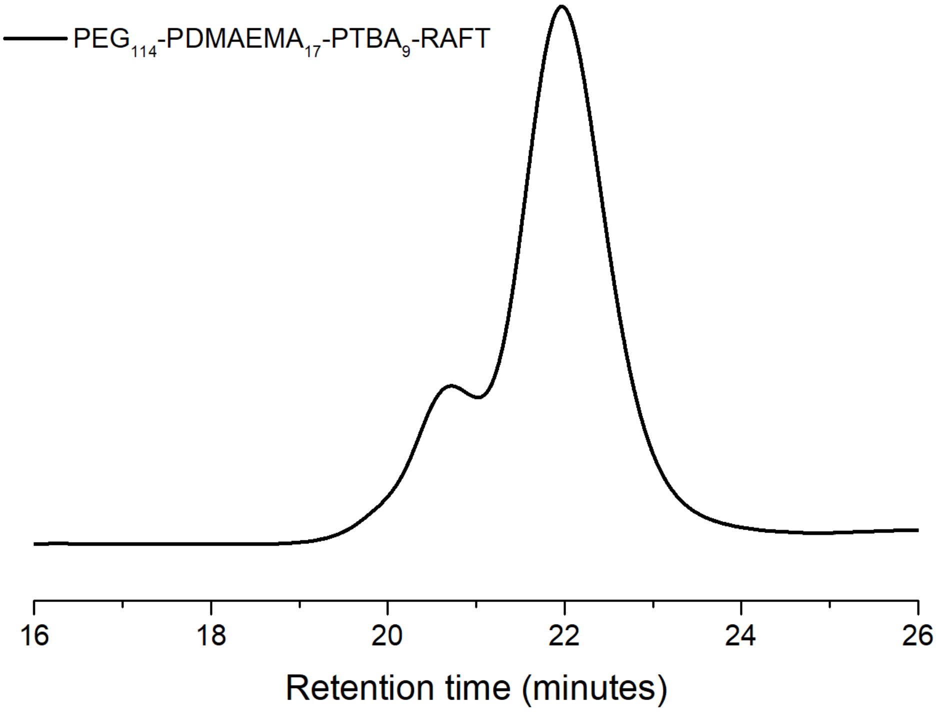

Figure S12. GPC trace of PEG_{114}-$PDMAEMA_{17}$-$PTBA_9$-RAFT (ABC6, M_n =15,300 g/mol, $Đ$ = 1.14) (DMF GPC, PMMA standards).

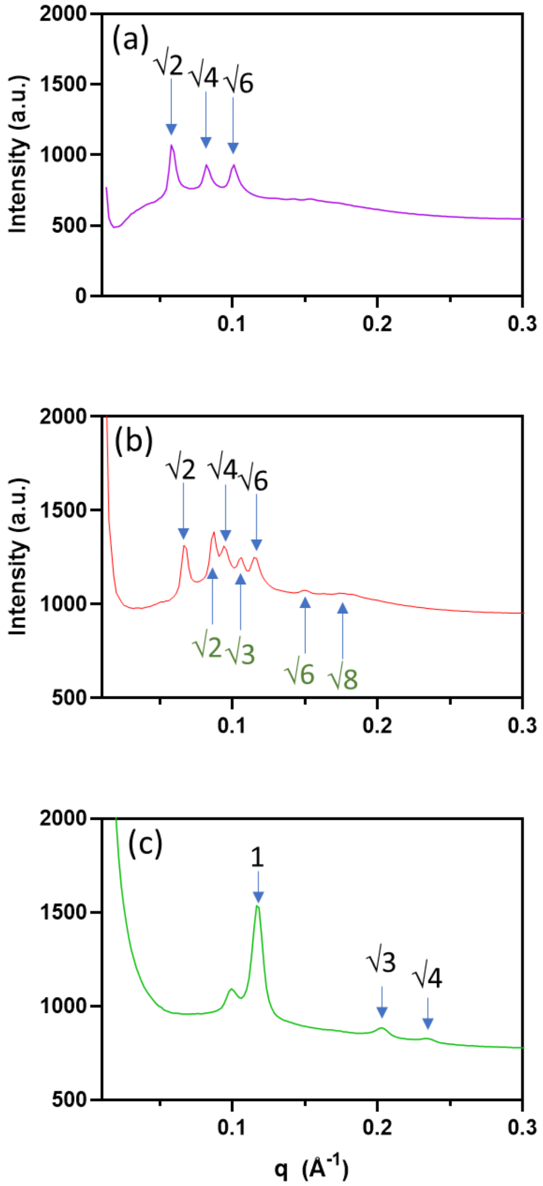

Figure S13. (a) The primitive cubic (Q_{II}^{P}) phase with symmetry group *Im3m* was identified by the peak spacing ratios of √2, √4, √6, illustrated by the sample stabilized with 0.5 mol% ABC3 at pH 7. (b) The sample stabilized with 0.5 mol% ABC5 at pH 7 exhibited a mixed cubic phase of Q_{II}^{P} as well as the double diamond cubic (Q_{II}^{D}) phase with symmetry group *Pn3m*, which was identified by the peak spacing ratios of √2, √3, √6, √8. (c) The sample stabilized with 3.0 mol% ABC4 at pH 7 exhibited a mixed Q_{II}^{D} phase (a small peak was visible) and the hexagonal (H_{II}) phase, which was identified by the peak spacing ratios of 1, √3, √4.

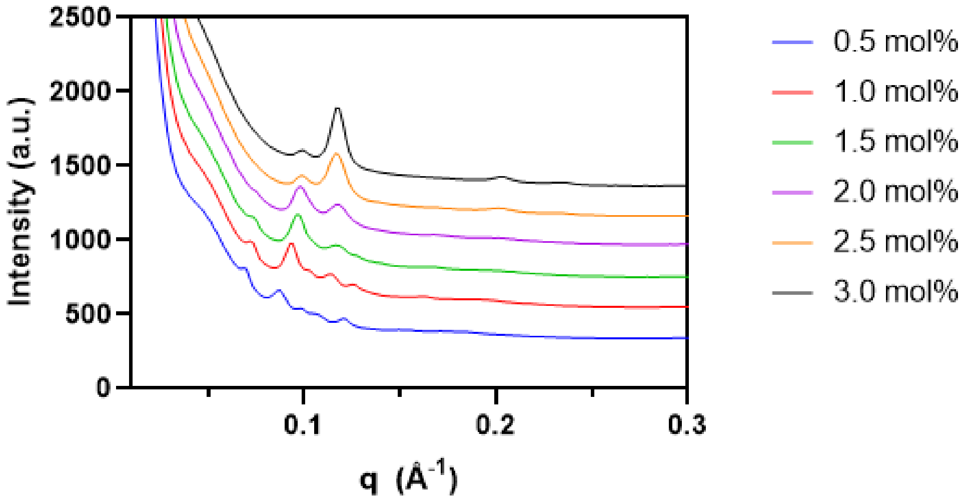

Figure S14. SAXS diffraction patterns of MO nanoparticles stabilized by ABC4 polymer at various concentrations after incubation with 50 mM H_2O_2 solution for one hour. Measurements were performed at 25 °C.

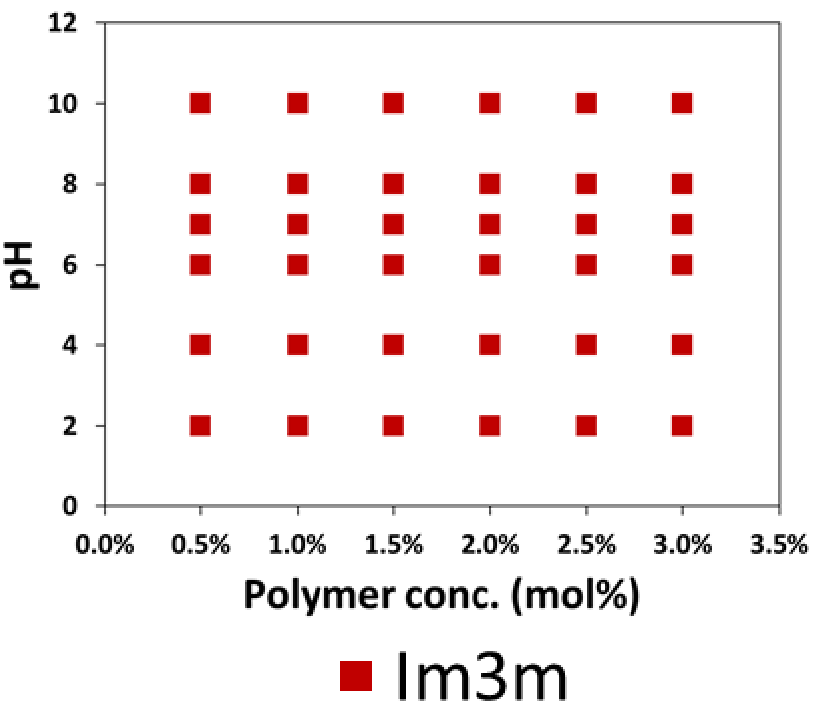

Figure S15. Mesophases of MO nanoparticles stabilized by ABC3 (the polymer without the pH-responsive PDMAEMA) at various concentrations in pH 2 to 10. All measurements were performed at 25 °C.
